# Supplementary material for: Treatment effect estimation by comparing observed and predicted outcomes: conditions for valid inference and practical illustration
Source: Diagn Progn Res. 2026 Jul 24;10:20. doi: 10.1186/s41512-026-00233-y (PMC13397745; doi:10.1186/s41512-026-00233-y)
Supplement: Supplementary file 1 — Supplementary Material 1. [file 41512_2026_233_MOESM1_ESM.pdf]

A Case study: illustration in R

A.1 Loading synthetic data

Here, we load the synthetic data that was generated as described in Appendix B. It contains 750 patients treated before the introduction of proton therapy, and 300 patients treated after the introduction of proton therapy, of whom 93 were treated with protons. It has the following columns:

variable	description
`proton_era`	Whether the patient was treated after the introduction of proton therapy
`treated_with_pt`	Whether the patient was treated with protons
`dys_m6_pht`	Presence of dysphagia grade >1 at 6 months post-radiotherapy with photons ($\sim Y_i(0)$). Empty for proton-treated patients
`dys_m6_prt`	Presence of dysphagia grade >1 at 6 months post-radiotherapy with protons ($\sim Y_i(1)$). Empty for photon-treated patients
`tumor_location`	Location of the tumor (larynx, oral cavity, or pharynx)
`dys_baseline`	Grade of dysphagia at baseline
`PCMsup_Dmean_pht`	Mean planned photon dose to the superior pharyngeal constrictor muscle
`PCMinf_Dmean_pht`	Mean planned photon dose to the inferior pharyngeal constrictor muscle
`PCMmed_Dmean_pht`	Mean planned photon dose to the middle pharyngeal constrictor muscle
`ORAL_ATL_Dmean_pht`	Mean planned photon dose to the oral cavity
`PCMsup_Dmean_prt`	Mean planned *proton* dose to the superior pharyngeal constrictor muscle
`PCMinf_Dmean_prt`	Mean planned *proton* dose to the inferior pharyngeal constrictor muscle
`PCMmed_Dmean_prt`	Mean planned *proton* dose to the middle pharyngeal constrictor muscle
`ORAL_ATL_Dmean_prt`	Mean planned *proton* dose to the oral cavity

The planned *proton* dose variables are only available for those patients treated after proton therapy was introduced.

```r
# loading necessary packages
require(dplyr)
require(table1)
require(ggplot2)
```

```r
# loading the data
pop = readRDS("synthetic_data.rds")

# inspecting a random sample of data
pop |> sample_n(5, random.seed=2)
```

```
##   proton_era treated_with_pt dys_m6_pht dys_m6_prt tumor_location
## 1          1               1         NA          1        Pharynx
## 2          0               0          0         NA        Pharynx
## 3          1               0          0         NA         Larynx
## 4          0               0          1         NA        Pharynx
## 5          0               0          0         NA        Pharynx
##                    dys_baseline PCMsup_Dmean_pht PCMsup_Dmean_prt
## 1     Grade 0-1 (regular diet)             69.2             63.7
## 2 Grade 3-5 (severe dysphagia)             57.4               NA
## 3     Grade 0-1 (regular diet)             20.9              4.9
## 4         Grade 2 (soft foods)             61.0               NA
## 5     Grade 0-1 (regular diet)             47.9               NA
##   PCMinf_Dmean_pht PCMinf_Dmean_prt PCMmed_Dmean_pht PCMmed_Dmean_prt
## 1             42.7             33.9             72.3             76.5
## 2             47.7               NA             46.1               NA
## 3             80.0             80.0             60.6             56.5
## 4             58.2               NA             62.0               NA
## 5             47.4               NA             30.3               NA
##   ORAL_ATL_Dmean_pht ORAL_ATL_Dmean_prt
## 1               55.2               47.3
## 2               46.0                 NA
## 3               21.3                9.4
## 4               56.0                 NA
## 5               37.3                 NA
```

A.2 Investigating positivity

We can check whether the covariate distributions in the proton-treated population seem to fall within the range of those observed in the pre-introduction population. As a pragmatic check, we examine the univariate distributions of the model covariates. Although this approach does not guarantee full support in the multivariate space, it is a pragmatic way to identify potential positivity violations.

```r
# selecting relevant patients
tab1_data = pop |>
  filter(!(proton_era == 1 & treated_with_pt == 0)) |>
  mutate(sample_cat =
           case_when(proton_era == 0 ~ # before introduction of protons
                       "Pre-introduction sample",
                     proton_era == 1 & treated_with_pt == 1 ~#treated with protons
                       "Proton-treated sample")
         )
```

First, we create a summary table of the patient characteristics. We do observe differences between the two populations, for example, in tumor location and planned dose distributions. However, there is overlap in the dose parameters, and tumor location categories and baseline dysphagia grades present in the proton-treated group are also represented in the pre-introduction sample.

```r
table1::table1(~ tumor_location + dys_baseline +
                 PCMsup_Dmean_pht + PCMinf_Dmean_pht +
                 PCMmed_Dmean_pht + ORAL_ATL_Dmean_pht | sample_cat,
               data=tab1_data,
               overall = NULL)
```

	Pre-introduction sample (N=750)	Proton-treated sample (N=93)
**tumor_location**		
Larynx	323 (43.1%)	5 (5.4%)
Oral cavity	48 (6.4%)	7 (7.5%)
Pharynx	379 (50.5%)	81 (87.1%)
**dys_baseline**		
Grade 0-1 (regular diet)	563 (75.1%)	59 (63.4%)
Grade 2 (soft foods)	126 (16.8%)	21 (22.6%)
Grade 3-5 (severe dysphagia)	61 (8.1%)	13 (14.0%)
**PCMsup_Dmean_pht**		
Mean (SD)	42.7 (22.5)	57.8 (13.4)
Median [Min, Max]	44.7 [0, 80.0]	57.8 [14.3, 80.0]
**PCMinf_Dmean_pht**		
Mean (SD)	54.5 (12.8)	49.7 (12.3)
Median [Min, Max]	55.0 [14.4, 80.0]	48.3 [20.2, 80.0]
**PCMmed_Dmean_pht**		
Mean (SD)	48.2 (18.9)	56.6 (14.3)
Median [Min, Max]	48.6 [0, 80.0]	56.7 [27.5, 80.0]
**ORAL_ATL_Dmean_pht**		
Mean (SD)	38.0 (21.1)	52.1 (13.1)
Median [Min, Max]	39.8 [0, 80.0]	51.8 [14.9, 78.8]

To further investigate the distributions of the continuous dose parameters, we can look at them in a boxplot. In the boxplot, we also observe that the distributions of the dosages seen in the proton-treated population seem to fall within the observed distributions of the pre-introduction population. Taken together, this suggests no concerns regarding the condition of positivity.

```r
tab1_data_long <- tab1_data |>
  tidyr::pivot_longer(cols = c(PCMsup_Dmean_pht,PCMinf_Dmean_pht,
                               PCMmed_Dmean_pht,ORAL_ATL_Dmean_pht),
                      names_to = "OAR", values_to = "dose")

ggplot(tab1_data_long, aes(y = dose, fill = sample_cat, color = sample_cat)) +
  geom_boxplot(alpha = 0.3) +
  theme_minimal() +
  labs(title = "Distribution of dose values by sample") +
  facet_wrap(~OAR)
```

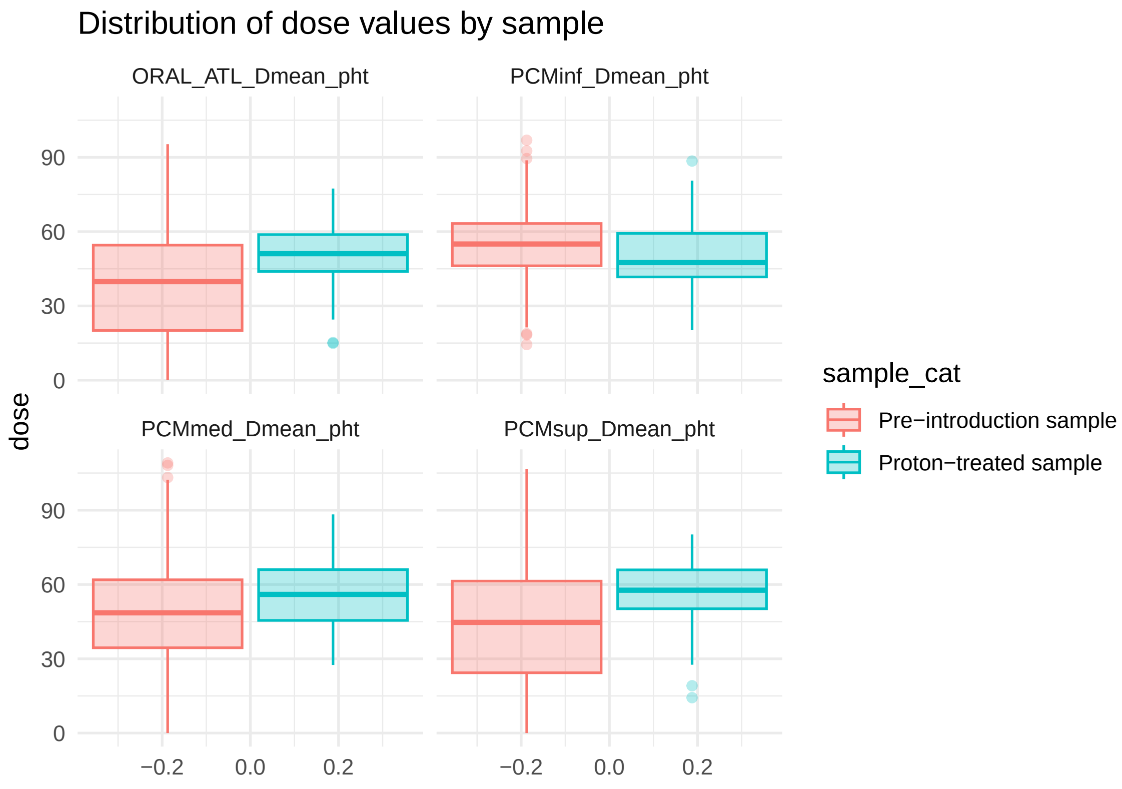

A.3 Fitting a model on pre-introduction data

Now, we start with estimating the treatment effect. Below, we first fit a logistic regression model on the patients treated before proton therapy was introduced, to predict grade >1 dysphagia at 6 months after radiotherapy.

As baseline patient characteristics $\mathbf{X}$ we use `tumor_location` and `dys_baseline`. As treatment plan variables (under the standard treatment) $\mathbf{Z}^{(0)}$ we use the photon dose plan variables: `PCMsup_Dmean_pht`, `PCMinf_Dmean_pht`, `PCMmed_Dmean_pht` and `ORAL_ATL_Dmean_pht`.

```
set.seed(2025)
# selecting the model development sample
pop_preintr = pop |> filter(proton_era==0)

# fit logistic regression model
mod = glm(dys_m6_pht ~
            # patient characteristics X
            tumor_location + dys_baseline +
            # photon dose parameters P^(0)
            ORAL_ATL_Dmean_pht + PCMsup_Dmean_pht +
            PCMmed_Dmean_pht + PCMinf_Dmean_pht,
```

```r
         data = pop_preintr,
         family = binomial())

# inspect model coefficients
mod |> broom::tidy() |>
  transmute(variable=term, coefficient = round(estimate, 3))
```

```
## # A tibble: 9 x 2
##   variable                                  coefficient
##   <chr>                                           <dbl>
## 1 (Intercept)                                    -4.98
## 2 tumor_locationOral cavity                       0.126
## 3 tumor_locationPharynx                          -0.45
## 4 dys_baselineGrade 2 (soft foods)                1.12
## 5 dys_baselineGrade 3-5 (severe dysphagia)        1.02
## 6 ORAL_ATL_Dmean_pht                              0.045
## 7 PCMsup_Dmean_pht                                0.034
## 8 PCMmed_Dmean_pht                                0.003
## 9 PCMinf_Dmean_pht                                0.007
```

A.4 Estimating the average treatment effect in the proton-treated population

Now that we have a model, we can apply it to the individuals treated with proton therapy, to predict what would have happened to them had they been treated with photons. This makes use of the fact that photon plan variables are available, even for patients that were treated with protons.

```r
# the patients that were treated with protons
pop_treated = pop |> filter(proton_era==1, treated_with_pt == 1)

# what we observed under protons
observed_outcomes = pop_treated$dys_m6_prt

# what we predict would have happened under photons
counterfactual_outcomes = predict(mod, pop_treated, type="response")

print(sprintf("Fraction of observed outcomes (under protons): %#.3f",
              mean(observed_outcomes)))
```

```
## [1] "Fraction of observed outcomes (under protons): 0.247"
```

```r
print(sprintf("Fraction of predicted outcomes (under photons): %#.3f",
              mean(counterfactual_outcomes)))
```

```
## [1] "Fraction of predicted outcomes (under photons): 0.468"
```

The ATT is calculated by calculating the average difference between the observed and predicted

outcomes:

$$\widehat{ATT} = \frac{1}{N} \sum_{i:T_i=1,\, i \in post} \left(Y_i - \mathbf{m}_{pre}(\mathbf{X}_i, \mathbf{Z}_i^{(0)}) \right)$$

where:

- Y_i is the observed outcome for patient i,
- $\mathbf{m}_{\text{pre}}(\mathbf{X}_i, \mathbf{Z}_i^{(0)})$ is the model-predicted outcome under photon therapy, given patient characteristics $\mathbf{X}_i$ and treatment plan details $\mathbf{Z}_i^{(0)}$.

```
print(sprintf("Average treatment effect in the treated: %#.3f",
              mean(observed_outcomes - counterfactual_outcomes)))
```

```
## [1] "Average treatment effect in the treated: -0.220"
```

A.5 Estimating confidence intervals using bootstrapping

We have estimated the ATT to be -0.22, i.e. an absolute risk reduction of dysphagia of 22%. However, even if all necessary assumptions hold, there is uncertainty around this estimate due to the finite sample size, which we estimate using bootstrapping. We bootstrap both the model development and prediction procedures.

First, we define the function that repeats the estimation procedure we saw above on bootstrapped samples of the data.

```
# function to estimate ATT using bootstrapping
bootstrap_att = function(pop_preintr,  # data frame with model development sample
                         pop_treated, # data frame with proton-treated target sample
                         observed_outcome_var, # name of observed outcome variable
                         n_boot = 1000,  # number of bootstrap replicates
                         seed = 2025) { # random seed for reproducibility

  set.seed(seed)

  # helper function: fits a logistic regression model
  # on 'train' and predicts on 'test'
  fit_predict = function(train, test) {
      model = glm(dys_m6_pht ~
                    tumor_location + dys_baseline +
                    ORAL_ATL_Dmean_pht + PCMsup_Dmean_pht +
                    PCMmed_Dmean_pht + PCMinf_Dmean_pht,
                  data = train,
                  family = binomial())
      predict(model, test, type = "response")
  }
```

```r
  # ----------- point estimate of ATT using full data -------------
  preds_full = fit_predict(pop_preintr, pop_treated)
  obs_full = pop_treated[[observed_outcome_var]]
  point_estimate = mean(obs_full) - mean(preds_full)

  # -------------- bootstrapped estimates of ATT ------------------
  # repeat the estimation procedure with bootstrapped samples
  boot_ests = replicate(n_boot, {
    boot_train = pop_preintr[sample(nrow(pop_preintr), replace = TRUE), ]
    boot_test  = pop_treated[sample(nrow(pop_treated), replace = TRUE), ]

    # fit and predict as before, but on bootstrapped sample
    preds = fit_predict(boot_train, boot_test)
    obs = boot_test[[observed_outcome_var]]

    # estimate ATT in this bootstrap sample
    mean(obs) - mean(preds)
  })

  # return both the point estimate and all bootstrap replicates
  return(list(
    point_estimate = point_estimate,
    boot_ests = boot_ests
  ))
}
```

Now that we have defined the function, we can apply it to our data.

```r
# apply function as defined above
att = bootstrap_att(pop_preintr, pop_treated,
                    observed_outcome_var = "dys_m6_prt")

# determine 95% CIs
sprintf("Estimated ATT: %#.2f, 95%% CI (%#.2f, %#.2f)",
      att$point_estimate,
      quantile(att$boot_ests, 0.025),
      quantile(att$boot_ests, 0.975))
```

```
## [1] "Estimated ATT: -0.22, 95% CI (-0.31, -0.13)"
```

We find a 95% confidence interval of (-0.31, -0.13). We see that the sampling uncertainty is quite high, but under the working model and bootstrap resampling scheme, the confidence interval excludes 0. This means that it is unlikely that the treatment is not beneficial (assuming all other conditions, e.g. transportability etc., hold).

A.6 Model validation in photon-treated sample (post-introduction)

To estimate the average treatment effect among the treated (ATT), we have made predictions about outcomes that are not observed and never will be. Specifically, for patients who received proton therapy, we estimated what would have happened if they had instead received photon therapy. As a result, it is fundamentally impossible to verify all the assumptions underlying our estimation procedure using observed data.

However, in some cases, auxiliary data can be used to provide indirect evidence for the plausibility of these assumptions. In our setting, even after the introduction of proton therapy, there were still patients who were treated with photons. These patients present an opportunity to assess the performance of our model: for them, we observe outcomes under photon therapy, and we can compare these observed outcomes to the model's predictions under the same treatment.

The photon-treated individuals act as a kind of negative control population. Instead of comparing observed outcomes under protons with predicted outcomes under protons (as in the ATT), we compare observed outcomes under photons with predicted outcomes under photons:

$$\frac{1}{N} \sum_{i:T_i=0, i \in \text{post}} \left(Y_i - \mathbf{m}_{\text{pre}}(\mathbf{X}_i, \mathbf{Z}_i^{(0)}) \right) \approx 0$$

Below, we calculate this difference between observed and predicted, which can also be recognized as a measure of model calibration (calibration-in-the-large).

```
# select 'negative controls' : patients treated with photons post-introduction
pop_nc = pop |> filter(proton_era==1, treated_with_pt == 0)

observed_outcomes = pop_nc$dys_m6_pht
predicted_outcomes = predict(mod, pop_nc, type="response")

print(sprintf("Fraction of observed outcomes (under photons): %#.2f",
              mean(observed_outcomes)))
```

```
## [1] "Fraction of observed outcomes (under photons): 0.25"
```

```
print(sprintf("Fraction of predicted outcomes (under photons): %#.2f",
              mean(predicted_outcomes)))
```

```
## [1] "Fraction of predicted outcomes (under photons): 0.25"
```

```
print(sprintf("Observed/expected absolute difference: %#.2f",
              mean(observed_outcomes - predicted_outcomes)))
```

```
## [1] "Observed/expected absolute difference: 0.00"
```

We now also estimate the uncertainty around this estimate, as before using bootstrapping. We can use the same function `bootstrap_att`, as the estimation procedure is exactly the same as for the ATT, except that the observed outcome variable is the outcome under photons instead.

```r
diff_observed_predicted = bootstrap_att(pop_preintr,
                                        pop_nc,
                                        observed_outcome_var = "dys_m6_pht")

# observed and predicted outcomes under photons are similar, with some uncertainty
sprintf("Estimated difference observed - predicted: %#.2f, 95%% CI (%#.2f, %#.2f)",
        diff_observed_predicted$point_estimate,
        quantile(diff_observed_predicted$boot_ests, 0.025),
        quantile(diff_observed_predicted$boot_ests, 0.975))
```

```
## [1] "Estimated difference observed - predicted: 0.00, 95% CI (-0.05, 0.05)"
```

We find that the difference is close to 0, which is also what we would expect if all assumptions hold. However, it is important to note that the converse does not necessarily hold: even if this check suggests good weak model calibration, it does not guarantee that the ATT estimates are unbiased, as these are based on a different subset of the post-introduction population (the proton-treated individuals). Nevertheless, a close correspondence between observed and predicted outcomes in the photon-treated group provides supportive evidence for the overall robustness of the estimation strategy.

Conversely, if a systematic difference would have been detected, such as an average underestimation of the outcome risk by 10%, this may indicate a violation of one or more assumptions. Several explanations are possible: it could be due to changes over time/setting (from model development to target population), or lack of ignorability of treatment assignment (the proton-treated group is systematically different from the photon-treated group, even after adjusting for measured covariates), or issues with model specification.

In such cases, one could choose to interpret the observed difference as being entirely due to a shift from model development to target population, i.e. a general temporal or geographical shift. Under this assumption, if the model tends to underestimate the risk of the outcome for patients treated with photon therapy, and we believe this same underestimation also applies to patients treated with protons, then the true benefit of proton therapy might be larger than what our model suggests. Vice versa: if the model overestimates the risk, the treatment effect might be smaller.

A.7 Model validation in proton-treated sample

Another opportunity to evaluate the model arises from the specific nature of the two treatments under comparison: both are forms of radiation therapy (photons and protons), and differ primarily in the amount of dose that is delivered.

If we assume that the relationship between planned radiation dose and outcome learned from photon therapy also applies to proton therapy, we can use this (extra) assumption to assess the validity of the model directly in the target population (i.e., patients treated with protons), even though the model was originally trained on photon-treated patients.

Let $\mathbf{Z}_i^{(1)}$ denote the planned proton dose parameters for patient i. We can then compare the observed outcomes under proton therapy to the predicted outcomes obtained by applying the photon-trained

model to these proton dose parameters:

$$\frac{1}{N}\sum_{i:T_i=1,i\in\text{post}}\left(Y_i-\mathbf{m}_{\text{pre}}(\mathbf{X}_i,\mathbf{Z}_i{}^{(1)})\right)\approx 0$$

If this difference approximates zero, and it is plausible that the dose-outcome relations are independent of treatment technology, this provides us further evidence of the robustness of the model. Again, the converse is not necessarily true: the model could give good predictions for the patients' outcomes under protons, but the outcomes under *photons* could be systematically off.

If a systematic difference is detected, this could again be due to various reasons: Again, it could be due to changes over time/setting, or lack of ignorability of treatment assignment, or issues with model specification. In this case, it could also be due to a systematic difference between proton and photon therapy, even after adjusting for dose plans (i.e., a certain amount of planned dose with photon therapy has a different effect than that same amount with proton therapy).

Again, one could decide to interpret a systematic difference as being due to differences between pre- and post-introduction population. In this case, if the risk of the outcomes under protons are overestimated, one could assume this would also hold for the outcomes under photons, in which case, the treatment effect would be overestimated (and vice versa).

Now, let's calculate the difference in observed and predicted outcomes (under protons) in our data set.

```
# select patients treated with protons in target population
pop_nc = pop |> filter(proton_era==1, treated_with_pt == 1)

# rename proton dose plan parameters to photon, to be able to apply the model
pop_nc_temp = pop_nc |>
                  dplyr::select(-ends_with("_pht")) |>
                  rename_with(~ stringr::str_replace(., "_prt$", "_pht"))

observed_outcomes = pop_nc$dys_m6_prt
predicted_outcomes = predict(mod, pop_nc_temp, type="response")

print(sprintf("Fraction of observed outcomes (under protons): %#.2f",
              mean(observed_outcomes)))
```

```
## [1] "Fraction of observed outcomes (under protons): 0.25"
```

```
print(sprintf("Fraction of predicted outcomes (under protons): %#.2f",
              mean(predicted_outcomes)))
```

```
## [1] "Fraction of predicted outcomes (under protons): 0.30"
```

```
print(sprintf("Observed/expected absolute difference: %#.2f",
              mean(observed_outcomes - predicted_outcomes)))
```

```
## [1] "Observed/expected absolute difference: -0.05"
```

```r
# now with uncertainty, again using the function defined above
diff_observed_predicted = bootstrap_att(pop_preintr,
                                        pop_nc_temp,
                                        # observed outcome was renamed above so
                                        # this refers to the outcome under protons
                                        observed_outcome_var = "dys_m6_pht")

sprintf("Estimated difference observed - predicted: %#.2f, 95%% CI (%#.2f, %#.2f)",
        diff_observed_predicted$point_estimate,
        quantile(diff_observed_predicted$boot_ests, 0.025),
        quantile(diff_observed_predicted$boot_ests, 0.975))
```

```
## [1] "Estimated difference observed - predicted: -0.05, 95% CI (-0.16, 0.05)"
```

We see that the predicted risk is overestimated: on average the predicted risk is around 5.4% higher than the observed risk. If we are willing to assume that this overestimation in outcomes under protons also applies to these same individuals had they received photons, then this would imply an *overestimation* of the treatment effect. However, this interpretation relies on other strong assumptions and the uncertainty is high due to limited data.

A.8 Sensitivity analysis: investigating the influence of estimator choice

So far, we have not addressed the assumption that the functional form of the model is correctly specified. While there are infinitely many ways to define the functional relationship between predictors and outcome, we can gain insight into the sensitivity of our results to these modeling choices by comparing the outcomes across several alternative model specifications. To get a rough idea, we compare three different models:

- Default logistic regression, no interactions
- GAM with splines for dose parameters
- XGBoost model, with default hyperparameters besides the number of iterations (trees)

```r
# loading additional packages
library(xgboost)
library(mgcv)
```

First, we create an extended version of the `bootstrap_att` function above, that allows us to vary the model choice.

```r
# an extended version of 'bootstrap_att', that allows us to vary the model type
bootstrap_att_extended = function(pop_preintr,# data frame model development sample
                                  pop_treated,# data frame proton-treated sample
                                  observed_outcome_var,# name of observed outcome
                                  predict_outcome_var,# name of variable to predict
                                  predictors,# list of predictors used
                                  model_type,# either 'glm', 'gam' or 'xgboost'
                                  n_boot = 1000,# number of bootstrap replications
```

```r
                                  seed = 2025# random seed for reproducibility
                                  ) {
set.seed(seed)

# helper function: fits a model of type 'model_type'
# on 'train' and predicts on 'test'
fit_predict = function(train, test) {
  if (model_type == "glm") {
    model_formula = as.formula(paste(predict_outcome_var, "~",
                               paste(predictors, collapse = " + ")))
    model = glm(model_formula, data = train, family = binomial())
    predict(model, test, type = "response")

  } else if (model_type == "gam") {
      spline_formula =  as.formula(dys_m6_pht ~ tumor_location + dys_baseline +
                                        s(ORAL_ATL_Dmean_pht) + s(PCMsup_Dmean_pht) +
                                        s(PCMmed_Dmean_pht) + s(PCMinf_Dmean_pht))
    model = gam(spline_formula, data = train, family = binomial())
    predict(model, test, type = "response")

  } else if (model_type == "xgboost") {
    x_train = model.matrix(~ . -1, data = train[, predictors])
    y_train = train[[predict_outcome_var]]
    dtrain = xgb.DMatrix(data = x_train, label = y_train)

    # determining optimal number of trees using cross-validation
    cv = xgb.cv(
      objective = "binary:logistic",
      eval_metric = "logloss",
      data = dtrain,
      nrounds = 1000, # maximum number
      nfold = 5,
      early_stopping_rounds = 15,
      verbose = 0
    )
    best_nrounds = cv$best_iteration

    # fitting final model
    model = xgb.train(
      objective = "binary:logistic",
      eval_metric = "logloss",
      data = dtrain,
      nrounds = best_nrounds,
      verbose = 0
    )
```

```
      x_test = model.matrix(~ . -1, data = test[, predictors])
      dtest = xgb.DMatrix(data = x_test)
      predict(model, newdata = dtest)
    }
  }

  # --------- point estimate of ATT using full data ---------
  preds_full = fit_predict(pop_preintr, pop_treated)
  obs_full = pop_treated[[observed_outcome_var]]
  point_estimate = mean(obs_full) - mean(preds_full)

  # --------- bootstrapped estimates of ATT -----------
  # repeat the estimation procedure with bootstrapped samples
  boot_ests = replicate(n_boot, {
    boot_train = pop_preintr[sample(nrow(pop_preintr), replace = TRUE), ]
    boot_test  = pop_treated[sample(nrow(pop_treated), replace = TRUE), ]

    # fit and predict as before
    preds = fit_predict(boot_train, boot_test)
    obs = boot_test[[observed_outcome_var]]

    # estimate ATT in this bootstrap sample
    mean(obs) - mean(preds)
  })

  # return both the point estimate and all bootstrap replicates
  return(list(
    point_estimate = point_estimate,
    boot_ests = boot_ests
  ))
}
```

We now apply this function to estimate the ATT with the different model choices.

```
predictors = c("tumor_location", "dys_baseline", "ORAL_ATL_Dmean_pht",
                "PCMsup_Dmean_pht", "PCMmed_Dmean_pht", "PCMinf_Dmean_pht")

# logistic regression
att_glm = bootstrap_att_extended(pop_preintr, pop_treated,
                                 observed_outcome_var = "dys_m6_prt",
                                 predict_outcome_var = "dys_m6_pht",
                                 predictors, model_type = "glm")

# GAM with splines
att_gam = bootstrap_att_extended(pop_preintr, pop_treated,
                                 observed_outcome_var = "dys_m6_prt",
```

```r
                                  predict_outcome_var = "dys_m6_pht",
                                  predictors, model_type = "gam")

# XGBoost (can be slow)
att_xgb = bootstrap_att_extended(pop_preintr, pop_treated,
                                  observed_outcome_var = "dys_m6_prt",
                                  predict_outcome_var = "dys_m6_pht",
                                  predictors, model_type = "xgboost")

# combine results in df
combined_results = list(
  glm = att_glm,
  gam = att_gam,
  xgboost = att_xgb
) |>
  purrr::imap_dfr(~{
    tibble(
      model = .y,
      point_estimate = .x$point_estimate,
      ci_lower = quantile(.x$boot_ests, 0.025),
      ci_upper = quantile(.x$boot_ests, 0.975)
    )
  })

combined_results
```

```
## # A tibble: 3 x 4
##   model   point_estimate ci_lower ci_upper
##   <chr>            <dbl>    <dbl>    <dbl>
## 1 glm             -0.220   -0.265   -0.116
## 2 gam             -0.215   -0.262   -0.135
## 3 xgboost         -0.230   -0.282   -0.144
```

Below, we plot the results of the different models.

```r
# plot
combined_results |> ggplot(aes(x = model, y = point_estimate)) +
  geom_point() +
  geom_errorbar(aes(ymin = ci_lower, ymax = ci_upper)) +
  labs(
    title = "Estimates by different modeling procedures",
    y = "ATT",
    x = "model"
  ) +
  geom_hline(yintercept = 0, linetype = "dashed") + # line of no effect
  theme_minimal()
```

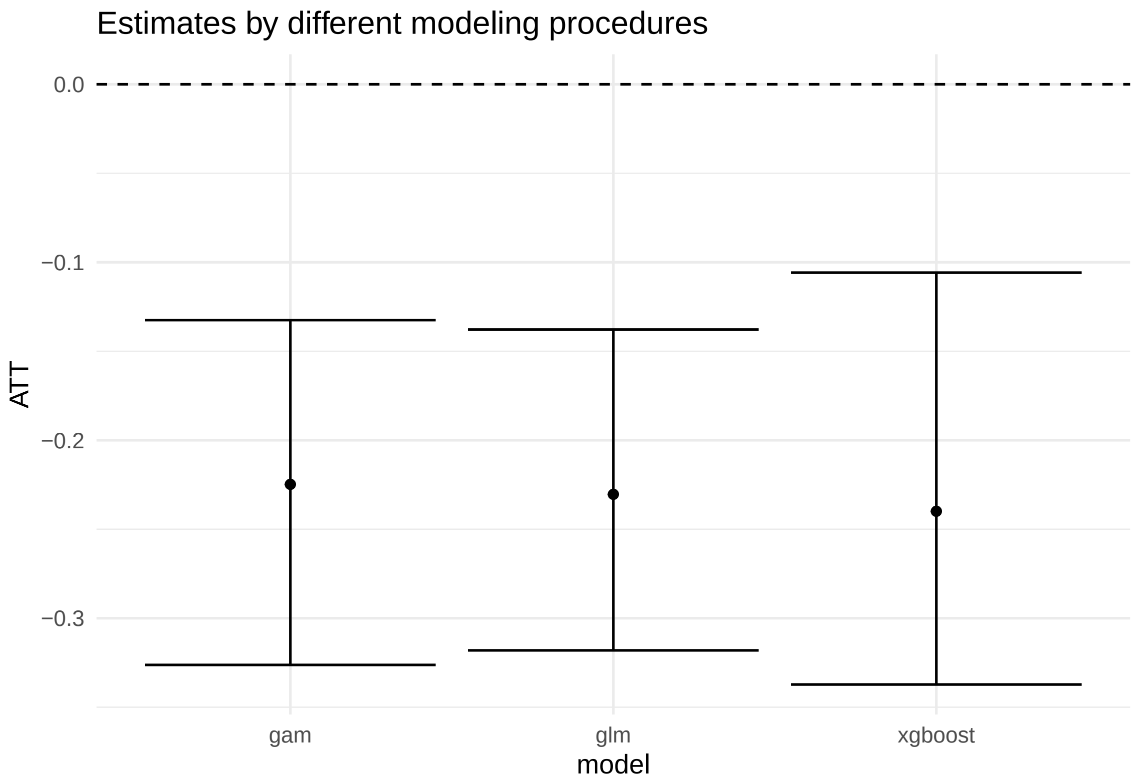

The results appear to be relatively stable across different modeling approaches, despite the fact that models like XGBoost are expected to learn very different predictor-outcome associations, especially with limited data. This suggests that the estimated effect is not highly sensitive to the choice of model. However, we do observe wider confidence intervals with `XGBoost`, which is expected given its greater flexibility and fewer assumptions compared to the more constrained `glm` model.

B Case study: generation of synthetic data

Tumor location We first draw tumor location

$$Tloc_i \in \{\text{Larynx},\ \text{Pharynx},\ \text{Oral Cavity}\}$$

from a categorical distribution

$$Tloc_i \sim \text{Categorical}\big(p_{\text{L}},\, p_{\text{P}},\, p_{\text{O}}\big),$$

with

$$(p_{\text{L}},\, p_{\text{P}},\, p_{\text{O}}) = (0.445,\ 0.496,\ 0.059).$$

Baseline dysphagia Conditional on $Tloc_i$, the baseline dysphagia grade $DysBase_i \in \{Grade\ 1, Grade\ 2, Grade\ 3-5\}$ is drawn as

$$DysBase_i \mid Tloc_i \ \sim\ \text{Categorical}\big(\pi_1(Tloc_i),\, \pi_2(Tloc_i),\, \pi_{3\text{–}5}(Tloc_i)\big),$$

where the log-odds are

$$\log\frac{\pi_2(Tloc)}{\pi_1(Tloc)} = -2.679 + 2.862\,\mathbf{1}\{Tloc = \text{Oral Cavity}\} + 1.556\,\mathbf{1}\{Tloc = \text{Pharynx}\},$$
$$\log\frac{\pi_{3\text{–}5}(Tloc)}{\pi_1(Tloc)} = -3.778 + 3.468\,\mathbf{1}\{Tloc = \text{Oral Cavity}\} + 2.101\,\mathbf{1}\{Tloc = \text{Pharynx}\}.$$

Planned photon doses Let $\mathbf{D}_i^{(ph)} = (D_{\text{ORAL}}, D_{\text{PCMsup}}, D_{\text{PCMmed}}, D_{\text{PCMinf}})_i^{\top}$. These planned photon dose parameters are drawn from a multivariate normal distribution:

$$\mathbf{D}_i^{(ph)} \sim \mathcal{N}_4\big(\mu_i^{(ph)}, \Sigma^{(ph)}\big),$$

with

$$\mu_i^{(ph)} = \beta_0 + \beta_1\,\mathbf{1}\{Tloc_i = \text{Oral Cavity}\} + \beta_2\,\mathbf{1}\{Tloc_i = \text{Pharynx}\},$$

where the intercept- and tumor location vectors are (e.g., the mean D_{ORAL} for patients with oral cavity tumor is $17.4 + 43.2$):

$$\beta_0 = \begin{pmatrix}17.4\\21.9\\37.2\\61.3\end{pmatrix}, \quad \beta_1 = \begin{pmatrix}43.2\\31.0\\13.1\\-18.8\end{pmatrix}, \quad \beta_2 = \begin{pmatrix}36.0\\38.7\\21.6\\-11.1\end{pmatrix}.$$

and the covariance matrix is

$$\Sigma^{(ph)} = \begin{pmatrix}164.44 & 175.28 & 156.47 & 13.57\\ 175.28 & 223.10 & 202.79 & 25.95\\ 156.47 & 202.79 & 302.09 & 101.75\\ 13.57 & 25.95 & 101.75 & 131.17\end{pmatrix}.$$

Finally we truncate negative doses and doses higher than 80: $\mathbf{D}_i^{(ph)} \leftarrow \max(\mathbf{D}_i^{(ph)}, 0)$ and $\mathbf{D}_i^{(ph)} \leftarrow \min(\mathbf{D}_i^{(ph)}, 80)$.

Planned proton doses Analogously, let $\mathbf{D}_i^{(pr)}$ be the proton dose vector $\mathbf{D}_i^{(pr)} = (D^{(pr)}_{\text{ORAL}}, D^{(pr)}_{\text{PCMsup}}, D^{(pr)}_{\text{PCMmed}}, D^{(pr)}_{\text{PCMinf}})_i^{\top}$. Its mean is now determined by tumor location and the corresponding photon dose parameter, i.e., the proton dose to the oral cavity is determined by the photon dose to the oral cavity, and the tumor location:

$$\mu_i^{(pr)} = \beta_0^{(pr)} + \beta_1^{(pr)}\,\mathbf{1}\{Tloc_i = \text{Oral Cavity}\} + \beta_2^{(pr)}\,\mathbf{1}\{Tloc_i = \text{Pharynx}\} + \beta_3^{(pr)}\,\mathbf{Z}_i,$$

where $\mathbf{Z}_i$ collects the corresponding organ-specific photon dose values, and

$$\beta_0^{(pr)} = \begin{pmatrix}-15.3\\-20.3\\-18.4\\-13.2\end{pmatrix}, \quad \beta_1^{(pr)} = \begin{pmatrix}0.088\\-0.174\\0.264\\-1.05\end{pmatrix}, \quad \beta_2^{(pr)} = \begin{pmatrix}-2.63\\1.30\\-0.52\\-2.23\end{pmatrix}, \quad \beta_3^{(pr)} = \begin{pmatrix}1.13\\1.23\\1.24\\1.17\end{pmatrix}.$$

For example, the mean proton dose to the oral cavity for patients with a tumor in the oral cavity, with a mean *photon* dose to the oral cavity of 30, would be $-15.3 + 0.088 + 1.13 \cdot 30$.

We then draw

$$\mathbf{D}_i^{(pr)} \sim \mathcal{N}_4\big(\mu_i^{(pr)}, \Sigma^{(pr)}\big),$$

With covariance matrix

$$\Sigma^{(pr)} = \begin{pmatrix} 24.24 & 7.27 & 2.84 & 2.92 \\ 7.27 & 21.59 & 7.89 & 3.31 \\ 2.84 & 7.89 & 24.51 & 9.31 \\ 2.92 & 3.31 & 9.31 & 21.96 \end{pmatrix}.$$

Cohort and treatment assignment We assign at random 750 patients to pre-introduction and 300 patients to post-introduction. In the post-introduction cohort, we compute for each i the predicted dysphagia risks using the logistic regression model currently used in the Dutch indication protocol for proton therapy for patients with head- and neck cancer [1]: $\hat{p}_i^{(ph)}$, $\hat{p}_i^{(pr)}$, and set

$$T_i = \begin{cases} 1 & \text{(proton) if } \hat{p}_i^{(ph)} - \hat{p}_i^{(pr)} > 0.10, \\ 0 & \text{(photon) otherwise.} \end{cases}$$

where

$$\begin{aligned} logit(\hat{p}_i^{(T_i)}) = &- 4.0536 + 0.9382\,\mathbf{1}\{DysBase_i = 2\} + 1.29\,\mathbf{1}\{DysBase_i = 3\text{–}5\} \\ &- 0.7711\,\mathbf{1}\{Tloc_i = \text{Larynx}\} - 0.6281\,\mathbf{1}\{Tloc_i = \text{Pharynx}\} \\ &+ 0.03\,D^{(T_i)}_{\text{ORAL,i}} + 0.0236\,D^{(T_i)}_{\text{PCMsup,i}} \\ &+ 0.0095\,D^{(T_i)}_{\text{PCMmed,i}} + 0.0133\,D^{(T_i)}_{\text{PCMinf,i}} \end{aligned}$$

Here $D^{(T_i)}$ denotes the dose vector under the modality that is evaluated. All pre-introduction patients have $T_i = 0$.

Outcome generation Finally, each patient's dysphagia outcome $DysM6_i \in \{0, 1\}$ is drawn from

$$DysM6 \sim \text{Bernoulli}\big(q_i\big),$$

with

$$\begin{aligned} logit(q_i) = &- 5.88 + 1.08\,\mathbf{1}\{DysBase_i = 2\} + 1.42\,\mathbf{1}\{DysBase_i = 3\text{–}5\} \\ &+ 0.40\,\mathbf{1}\{Tloc_i = \text{Oral Cavity}\} + 0.17\,\mathbf{1}\{Tloc_i = \text{Pharynx}\} \\ &+ 0.07\,D^{(T_i)}_{\text{ORAL,i}} - 0.0003\,D^{(T_i)}_{\text{PCMsup,i}} \\ &+ 0.016\,D^{(T_i)}_{\text{PCMmed,i}} + 0.018\,D^{(T_i)}_{\text{PCMinf,i}} \end{aligned}$$

Here $D^{(T_i)}$ denotes the dose vector under the assigned modality.

C Derivation from estimand to estimator

Below, we demonstrate how the assumptions of transportability, ignorability of treatment assignment, positivity, consistency, and correct model specification jointly enable the identification of the causal estimand (formulated under the potential outcomes framework) from observed data via a model-based estimator.

$$
\begin{aligned}
ATT &= \mathbb{E}_{post}[Y(1) - Y(0) \mid T = 1] && (1)\\
&= \mathbb{E}_{post}[Y(1) \mid T = 1] - \mathbb{E}_{post}[Y(0) \mid T = 1] && (2)\\
&= \mathbb{E}_{post}[Y(1) \mid T = 1] - \mathbb{E}_{post}\left[\mathbb{E}_{post}[Y(0) \mid \mathbf{X}, \mathbf{Z}^{(0)}, T = 1]\middle| T = 1\right] && (3)\\
&= \mathbb{E}_{post}[Y(1) \mid T = 1] - \mathbb{E}_{post}\left[\mathbb{E}_{post}[Y(0) \mid \mathbf{X}, \mathbf{Z}^{(0)}]\middle| T = 1\right] && (4)\\
&= \mathbb{E}_{post}[Y(1) \mid T = 1] - \mathbb{E}_{post}\left[\mathbb{E}_{pre}[Y(0) \mid \mathbf{X}, \mathbf{Z}^{(0)}]\middle| T = 1\right] && (5)\\
&= \mathbb{E}_{post}[Y \mid T = 1] - \mathbb{E}_{post}\left[\mathbb{E}_{pre}[Y \mid \mathbf{X}, \mathbf{Z}^{(0)}]\middle| T = 1\right] && (6)\\
&= \mathbb{E}_{post}[Y \mid T = 1] - \mathbb{E}_{post}\left[\mathbf{m}_{pre}(\mathbf{X}, \mathbf{Z}^{(0)}; \boldsymbol{\beta}_\mathbf{0})\middle| T = 1\right] && (7)\\
&\approx \frac{1}{N} \sum_{i:T_i=1,\, i\in post} Y_i - \frac{1}{N} \sum_{i:T_i=1,\, i\in post} \mathbf{m}_{pre}(\mathbf{X}_i, \mathbf{Z}_i^{(0)}; \hat{\boldsymbol{\beta}}) && (8)\\
&= \frac{1}{N} \sum_{i:T_i=1,\, i\in post} \left(Y_i - \mathbf{m}_{pre}(\mathbf{X}_i, \mathbf{Z}_i^{(0)}; \hat{\boldsymbol{\beta}})\right) && (9)
\end{aligned}
$$

Explanation of steps:

- (1): Average treatment effect among the treated in the post-introduction population, defined in potential outcomes, as described in section 3.1.
- (2): By linearity of expectation.
- (3): Using the law of total expectation.
- (4): Assuming ignorability of treatment assignment given $\mathbf{X}$ and $\mathbf{Z}^{(0)}$, as described in section 3.3.2.
- (5): Assuming transportability (section 3.3.1) and positivity (section 3.3.4).
- (6): Under consistency (section 3.3.3): observed outcomes correspond with the potential outcomes under the realized treatment (which is always T=0 in the pre-introduction population).
- (7): There exists a correct model fit $\mathbf{m}_{pre}(X, Z^0; \boldsymbol{\beta}_\mathbf{0}) = \mathbb{E}_{pre}[Y \mid \mathbf{X}, \mathbf{Z}^{(0)}]$ (section 3.3.5).
- (8): Using an empirical approximation of the expectations, by averaging over a sample of the target treatment population, under standard likelihood theory.

- (9): Simplified final form showing ATT as the average difference between actual and model-predicted counterfactuals in a sample from the treated target population.

These conditions are sufficient but not strictly necessary. For example, the transition from equation (3) to (5) does not require full ignorability of treatment assignment or full transportability. It is sufficient that the conditional distributions of potential outcomes under standard treatment is stable from pre-introduction population to the target treatment population:

$$\mathbb{E}_{post}[Y(0) \mid \mathbf{X}, \mathbf{Z}^{(0)}, T = 1] = \mathbb{E}_{pre}[Y(0) \mid \mathbf{X}, \mathbf{Z}^{(0)}].$$

In other words, it is not necessary that the distribution of potential outcomes patients with assigned treatment $T = 0$ post-introduction match those in the pre-introduction period, only that the conditional expectation remains invariant within the relevant subpopulation (the target treatment population).

Ultimately, what matters is whether the estimator

$$\frac{1}{N} \sum_{i:T_i=1,\, i \in post} \mathbf{m}_{pre}(\mathbf{X}_i, \mathbf{P}_i^{(0)}; \hat{\boldsymbol{\beta}})$$

is a good approximation of the counterfactual mean $\mathbb{E}_{post}[Y(0) \mid T = 1]$. This implies that even if the model $\mathbf{m}_{pre}$ is misspecified, or if the conditional distributions shift slightly between periods, unbiased estimation of the ATT may still happen provided that errors average out.

D Case study: graphical representation

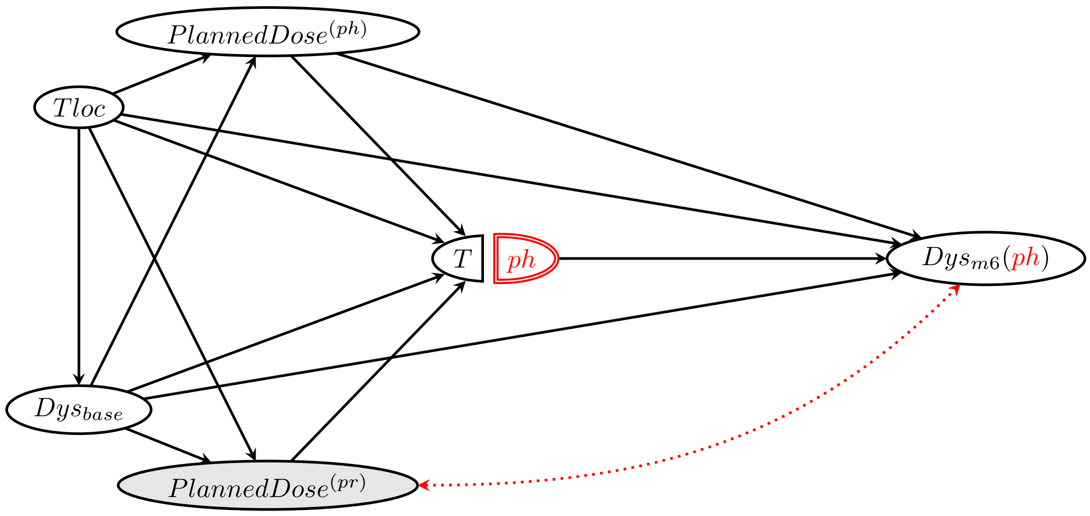

Figure 1: Graphical representation of the assumed causal structure underlying the case study. The red dotted arrow indicates a potential unmeasured confounding path between the planned dose variables under proton therapy (which are unmeasured in the model development population) and the outcome Y under photon therapy. Such confounding would violate the ignorability of treatment assignment assumption by introducing a non-causal association between the treatment assignment and the outcome, not attributable to the effect of the treatment itself.

We represent the assumed causal structure of the problem in Figure 1 using a Single World Intervention Graph (SWIG), as introduced by Richardson and Robins [2]. This graphical tool allows us to explicitly focus on the potential outcome of interest: dysphagia at six months under photon therapy, denoted $Dys_{m6}(ph)$. Since our analysis aims to estimate what would have happened to all patients had they received photons, this SWIG formulation is more suitable than a standard directed acyclic graph (DAG). A defining feature of the SWIG is that the treatment node T is split into two components. The left part of the node represents the naturally assigned treatment, i.e., the treatment selected for each patient by model-based selection. The arrows pointing into this node indicate that treatment assignment is a function of baseline dysphagia, tumor location, planned dose variables under photon therapy, and planned dose variables under proton therapy. These arrows capture our assumptions about which pre-treatment variables influenced the treatment selection.

The right part of the split node represents the intervened treatment value, here fixed to photon therapy (ph). This reflects the hypothetical scenario where all patients receive photons, and we examine the outcome under this intervention. The right-hand side is connected to the outcome node $Dys_{m6}(ph)$ to indicate that we are studying the potential outcome under this intervention.

The outcome is assumed to be causally influenced by baseline dysphagia, tumor location, and planned dose parameters under photon therapy. These variables have arrows pointing into the outcome node. Variables not connected by arrows are assumed not to have direct causal effects on one another. Likewise, variables absent from the graph are assumed either not to influence any other variables in

the graph or only to influence a single variable, in which case they are not sources of confounding.

The planned proton dose variables is visualized greyed-out, to indicate that this variable is unmeasured (unaccounted for) in the analysis, as it is not incorporated in the model used to make the counterfactual predictions.

This representation of the case study helps us see that if there is confounding of the planned dose under protons and the outcome under photons, this would result in a violation of the ignorability of treatment assignment assumption (indicated by the red dotted arrow). For example, if there is an unmeasured variable that influences both the planned dose under protons and the outcome under photons, this could result in bias. An example of such a variable may be a patients' N-stage. By contrast, the other determinants of treatment assignment—baseline dysphagia, tumor location, and planned photon dose—are observed and included as covariates in the model, and therefore do not pose a threat to ignorability.

This SWIG captures the causal relationships in the post-introduction setting, where both photon and proton therapies are available. Its main purpose is to support reasoning about whether treatment assignment can be considered ignorable, given observed covariates. While this graph focuses on ignorability, it could be extended to address issues of transportability, e.g. following the framework of Bareinboim and Pearl [3].

References

[1] *Landelijk Indicatie Protocol Protonen Therapie Hoofdhals versie 2.2.* 2019. URL: https://nvro.nl/images/documenten/rapporten/2019-08-15___Landelijk_Indicatieprotocol_Protonentherapie_Hoofdhals_v2.2.pdf (visited on 05/28/2025).

[2] Thomas S. Richardson and James M. Robins. *Single world intervention graphs (SWIGs): A unification of the counterfactual and graphical approaches to causality.* Tech. rep. 128. Working paper. Center for Statistics and the Social Sciences, University of Washington, 2013. URL: https://csss.uw.edu/research/working-papers/single-world-intervention-graphs-swigs-unification-counterfactual-and.

[3] Elias Bareinboim and Judea Pearl. "Causal inference and the data-fusion problem". In: *Proceedings of the National Academy of Sciences* 113.27 (July 2016). Publisher: Proceedings of the National Academy of Sciences, pp. 7345–7352. DOI: 10.1073/pnas.1510507113.
